# Author Correction: Neural and molecular changes during a mind-body reconceptualization, meditation, and open label placebo healing retreat: an observational study

**DOI:** 10.1038/s42003-026-10719-6

**Published:** 2026-07-25

**Authors:** Alex Jinich-Diamant, Sierra Simpson, Juan P. Zuniga-Hertz, Ramamurthy Chitteti, Jan M. Schilling, Jacqueline A. Bonds, Laura Case, Andrei V. Chernov, Joe Dispenza, Jacqueline Maree, Natalia Esther Amkie Stahl, Michael Licamele, Narin Fazlalipour, Swetha Devulapalli, Leonardo Christov-Moore, Nicco Reggente, Michelle A. Poirier, Tobias Moeller-Bertram, Hemal H. Patel

**Affiliations:** 1 Department of Anesthesiology, University of California San Diego, La Jolla, CA USA; 2 Department of Cognitive Science, University of California San Diego, La Jolla, CA USA; 3 Veterans Affairs San Diego Healthcare System, La Jolla, CA USA; 4 Metamorphosis LLC, Ranier, WA USA; 5 VitaMed Research, Palm Desert, CA USA; 6 Institute for Advanced Consciousness Studies, Santa Monica, CA USA

**Keywords:** Cognitive neuroscience, Proteomics, Metabolomics, Cellular neuroscience

Correction to: *Communications Biology* 10.1038/s42003-025-09088-3, published online 06 November 2025

Following publication of this article, the title has been updated to clarify that the study was observational rather than interventional, with text added in the Results and Methods to specify which analyses were pre-registered. The competing interests statement has also been updated to clarify the competing interests of Joe Dispenza and state those of Hemal H. Patel. The changes are made in the HTML and PDF versions of the article.

