# Supplementary material for: Neural and molecular changes during a mind-body reconceptualization, meditation, and open label placebo healing retreat: an observational study
Source: Commun Biol. 2025 Nov 6;8:1525. doi: 10.1038/s42003-025-09088-3 (PMC12592435; doi:10.1038/s42003-025-09088-3)
Supplement: Supplementary file 2 — Supplementary Figs. and Tables [file 42003_2025_9088_MOESM2_ESM.pdf]

Supplementary Tables

Supplementary Table 1: fMRI Framewise Displacement

	Pre		Post	
**Task**	***M***	***SD***	***M***	***SD***
meditation	0.157	0.113	0.140	0.121
rest	0.086	0.095	0.088	0.096

Supplementary Table 2: fMRI Two-Way (Scan X Time) Mean Framewise Displacement Repeated Measures ANOVA

**Source**	***SS***	***DF1***	***DF2***	***MS***	***F***	***p***	η^2p
scan	0.072	1	18	0.072	25.1	0.0001	0.58
time	0.001	1	18	0.001	0.6	0.44	0.03
scan * time	0.002	1	18	0.002	1.3	0.26	0.07

Supplementary Table 3: Montreal Neurologic Institute (MNI) Coordinates for 36 Regions of Interest Making up 7 Resting State Networks (source: Raichle 2011[91])

		MNI Coordinate		
**Region of Interest**	**Network**	***x***	***y***	***z***
dorsal medial PFC	ECN	0	24	46
left anterior PFC	ECN	-44	45	0
right anterior PFC	ECN	44	45	0
left superior parietal	ECN	-50	-51	45
right superior parietal	ECN	50	51	45
PCC	DMN	0	-52	27
mPFC	DMN	-1	54	27
left lateral parietal	DMN	-46	-66	30
right lateral parietal	DMN	49	-63	33
left inferior temporal	DMN	-61	-24	-9
right inferior temporal	DMN	58	-24	-9

medial dorsal thalamus	DMN	0	-12	9
right posterior cerebellum	DMN	-25	-81	-33
left posterior cerebellum	DMN	25	-81	-33
left frontal eye field	DAN	-29	-9	54
right frontal eye field	DAN	29	-9	54
left posterior IPS	DAN	-26	-66	48
right posterior IPS	DAN	26	-66	48
left anterior IPS	DAN	-44	-39	45
right anterior IPS	DAN	41	-39	45
left MT	DAN	-50	-66	-6
right MT	DAN	53	-63	-6
dorsal ACC	SN	0	21	36
left anterior PFC	SN	-35	45	30
right anterior PFC	SN	32	45	30
left insula	SN	-41	3	6
right insula	SN	41	3	6
left lateral parietal	SN	-62	-45	30
right lateral parietal	SN	62	-45	30
left motor cortex	SMN	-39	-26	51
right motor cortex	SMN	38	-26	48
SMA	SMN	0	-21	48
left V1	VN	-7	83	2
right V1	VN	7	83	2
left A1	AN	-62	-30	12
right A1	AN	59	-27	15

PFC: prefrontal cortex; PCC: posterior cingulate cortex; IPS: intraparietal sulcus; MT: middle temporal region; ACC: anterior cingulate cortex; SMA: supplementary motor area; V1: primary visual cortex; A1: primary auditory cortex; ECN: executive control network; DMN: default mode network; DAN: dorsal attention network; SN: salience network; SMN: somatomotor network; VN: visual network; AN: auditory network.

Supplementary Table 4: Montreal Neurologic Institute (MNI) Coordinates for 8 *a priori* Regions of Interest (DiFuMo atlas)

	**MNI Coordinate**		
**Region of Interest**	***x***	***y***	***z***
left dlPFC	-6	44	-6
right dlPFC	52	-52	32

left anterior insula	-50	-56	30
right anterior insula	-3	-42	25
central precuneus	38	20	-2
left angular gyrus	-34	16	2
right angular gyrus	44	36	32
mPFC	-44	34	32

dlPFC: dorsolateral prefrontal cortex; mPFC: medial prefrontal cortex.

Supplementary Table 5. Statistically Significant ($p < 0.05$) Rest-vs-Meditation Paired t-tests Between A Priori Regions of Interest

Session	A Priori Region Pair		*t*	*p*	Cohen's *d*
pre	L angular gyrus	precuneus	-2.12	0.05	-0.66
pre	R angular gyrus	R dlPFC	-2.69	0.02	-0.58
pre	mPFC	precuneus	-2.70	0.02	-0.85
pre	mPFC	L angular gyrus	-3.72	0.002	-1.08
pre	mPFC	R angular gyrus	-2.49	0.02	-0.90
post	L anterior insula	L dlPFC	-2.15	0.05	-0.68
post	R anterior insula	L dlPFC	-2.27	0.04	-0.65
post	R anterior insula	L anterior insula	-2.21	0.04	-0.78
post	L angular gyrus	precuneus	-2.72	0.01	-0.75
post	R angular gyrus	precuneus	-2.63	0.02	-0.78
post	R angular gyrus	L angular gyrus	-3.09	0.01	-0.97
post	mPFC	precuneus	-2.20	0.04	-0.70
post	mPFC	L angular gyrus	-3.34	0.004	-1.00
post	mPFC	R angular gyrus	-3.79	0.001	-1.05

dlPFC: dorsolateral prefrontal cortex; mPFC: medial prefrontal cortex.

Supplementary Table 6: Whole-Brain Functional Connectivity Network Measures

		Rest				Meditation			
		Pre		Post		Pre		Post	
Atlas	Measure	*M*	*SD*	*M*	*SD*	*M*	*SD*	*M*	*SD*
Harvard-Oxford	Characteristic Path Length	0.0243	0.0167	0.0263	0.0263	0.0240	0.0307	0.0131	0.0106

	Global Efficiency	-0.0009	0.0002	-0.0009	0.0003	-0.0006	0.0002	-0.0006	0.0002
	Modularity	0.0394	0.0039	0.0373	0.0044	0.0352	0.0039	0.0332	0.0059
Power	Characteristic Path Length	0.0882	0.0591	0.0996	0.0539	0.0808	0.0111	0.0562	0.0183
	Global Efficiency	-0.00035	0.00001	-0.00003	0.00001	-0.00003	0.00005	-0.00002	0.000003
	Modularity	0.0387	0.0045	0.0380	0.0029	0.0307	0.0062	0.0284	0.0035

Supplementary Table 7: 2 (Time: Pre/Post) by 2 (Task: Rest/Meditation) Repeated Measures ANOVA on Whole-Brain Modularity, Characteristic Path Length, and Global Efficiency

Atlas	Measure	Source	*SS*	*DF1*	*DF2*	*MS*	*F*	*p*	*η²p*
Harvard-Oxford	Modularity	task	0.00032	1	18	0.0003	15	**0.001**	0.45
		session	7.8e-5	1	18	7.8e-5	3	0.098	0.14
		task * sess.	2.7e-8	1	18	2.7e-8	0.001	0.97	6.5e-5
	Char. Path Length	task	0.00087	1	18	0.00087	1.7	0.21	0.09
		session	0.00038	1	18	0.00038	0.9	0.35	0.05
		task * sess.	0.00078	1	18	0.00078	1.5	0.23	0.08
	Global Efficiency	task	1.8e-6	1	18	1.8e-6	48	**0.000002**	0.73
		session	2.5e-9	1	18	2.5e-9	0.07	0.80	0.004
		task * sess.	2.8e-9	1	18	2.8e-9	0.11	0.75	0.006
Power	Modularity	task	0.0014	1	17	0.0014	64	**3.6e-07**	0.79
		session	4e-05	1	17	4e-05	1.6	0.22	0.09
		task * sess.	1.1e-5	1	17	1.1e-05	0.53	0.48	0.03
	Char. Path Length	task	0.012	1	17	0.012	2.6	0.12	0.13
		session	0.0008	1	17	0.0008	0.15	0.70	0.009
		task * sess.	0.0058	1	17	0.0058	1.5	0.23	0.08
	Global Efficiency	task	3.1e-09	1	17	3.1e-09	4.8	**0.042**	0.22
		session	8.9e-10	1	17	8.9e-10	1.5	0.23	0.083
		task * sess.	6.2e-10	1	17	6.2e-10	0.9	0.36	0.05

Bold denotes statistical significance ($p < 0.05$).

Supplementary Table 8: Pre- vs. Post-Intervention *a Priori* Regions Functional Connectivity

Task	A Priori Region Pair		*t*	*p*	Cohen's *d*
rest	right angular gyrus	mPFC	-0.09	0.93	-0.02
rest	left angular gyrus	mPFC	1.08	0.29	0.30
rest	left angular gyrus	right angular gyrus	0.19	0.85	0.04
rest	central precuneus	mPFC	0.40	0.70	0.12
rest	central precuneus	right angular gyrus	-1.16	0.26	-0.31

rest	central precuneus	left angular gyrus	-0.22	0.83	-0.08
rest	right anterior insula	mPFC	0.91	0.38	0.27
rest	right anterior insula	right angular gyrus	-1.16	0.26	-0.25
rest	right anterior insula	left angular gyrus	-1.24	0.23	-0.34
rest	right anterior insula	central precuneus	-0.58	0.57	-0.16
rest	left anterior insula	mPFC	-0.22	0.82	-0.07
rest	left anterior insula	right angular gyrus	-0.79	0.44	-0.20
rest	left anterior insula	left angular gyrus	-0.44	0.66	-0.14
rest	left anterior insula	central precuneus	0.11	0.92	0.03
rest	left anterior insula	right anterior insula	0.51	0.62	0.17
rest	right dlPFC	mPFC	0.49	0.63	0.12
rest	right dlPFC	right angular gyrus	-1.04	0.31	-0.32
rest	right dlPFC	left angular gyrus	-0.07	0.95	-0.02
rest	right dlPFC	central precuneus	-1.01	0.33	-0.27
rest	right dlPFC	right anterior insula	1.06	0.30	0.33
rest	right dlPFC	left anterior insula	1.65	0.12	0.57
rest	left dlPFC	mPFC	-0.54	0.60	-0.13
rest	left dlPFC	right angular gyrus	-0.14	0.89	-0.04
rest	left dlPFC	left angular gyrus	0.72	0.48	0.16
rest	left dlPFC	central precuneus	0.88	0.39	0.21
rest	left dlPFC	right anterior insula	-0.78	0.45	-0.20
rest	left dlPFC	left anterior insula	0.31	0.76	0.09
rest	left dlPFC	right dlPFC	-0.09	0.93	-0.02
meditation	right angular gyrus	mPFC	-0.10	0.93	-0.02
meditation	left angular gyrus	mPFC	0.07	0.95	0.02
meditation	left angular gyrus	right angular gyrus	-1.37	0.19	-0.38
meditation	central precuneus	mPFC	-0.02	0.98	-0.01
meditation	central precuneus	right angular gyrus	-0.99	0.34	-0.24
meditation	central precuneus	left angular gyrus	-0.40	0.69	-0.12
meditation	right anterior insula	mPFC	-0.07	0.94	-0.02
meditation	right anterior insula	right angular gyrus	-0.30	0.77	-0.09
meditation	right anterior insula	left angular gyrus	-0.37	0.72	-0.12
meditation	right anterior insula	central precuneus	-0.73	0.48	-0.22
meditation	left anterior insula	mPFC	0.69	0.50	0.19
meditation	left anterior insula	right angular gyrus	0.06	0.95	0.02
meditation	left anterior insula	left angular gyrus	0.20	0.84	0.06
meditation	left anterior insula	central precuneus	-0.75	0.46	-0.22
meditation	left anterior insula	right anterior insula	0.42	0.68	0.12
meditation	right dlPFC	mPFC	-1.24	0.23	-0.35
meditation	right dlPFC	right angular gyrus	-1.01	0.33	-0.29
meditation	right dlPFC	left angular gyrus	-1.15	0.27	-0.30

meditation	right dlPFC	central precuneus	-1.23	0.23	-0.28
meditation	right dlPFC	right anterior insula	-1.00	0.33	-0.29
meditation	right dlPFC	left anterior insula	-1.09	0.29	-0.34
meditation	left dlPFC	mPFC	-1.27	0.22	-0.30
meditation	left dlPFC	right angular gyrus	-0.66	0.52	-0.23
meditation	left dlPFC	left angular gyrus	-0.04	0.97	-0.01
meditation	left dlPFC	central precuneus	-0.99	0.34	-0.30
meditation	left dlPFC	right anterior insula	-0.75	0.46	-0.27
meditation	left dlPFC	left anterior insula	0.13	0.90	0.04
meditation	left dlPFC	right dlPFC	-0.49	0.63	-0.17

dlPFC: dorsolateral prefrontal cortex; mPFC: medial prefrontal cortex.

Supplementary Table 9. Significant Anatomical Gray Matter Volume Clusters for Advanced > Novice Contrast

Location	Vertices (#)	Cluster (*p*)	Peak MNI (x, y, z)	Group diff. (peak *p*)	Cluster surface (mm^2)
Superior parietal lobule	269	0.049	19.8, -66.3, 51.0	0.00027	166.33

Supplementary Table 10: fMRI Meditation Depth and Expectations Survey Results

	Pre		Post	
	M	*SD*	*M*	*SD*
How well/deeply did you meditate (1 to 10)?	6.05	1.75	6.89	1.91
Meditation depth compared to recent meditation experience (-5 to 5).	-0.11	2.85	-0.32	2.71
Retreat expectation (pre) and perceived effect (post) on meditation ability.	8.95	1.47	8.74	1.79

Supplementary Table 11. ELISA Biomarker Levels and Wilcoxon Signed Rank Test (Pre- vs Post-Intervention)

	Pre		Post		Pre vs. Post	
ELISA	*M*	*SD*	*M*	*SD*	*p*	Cohen's d
CART (pg/ml)	413.34	497.1	395.32	363.22	0.37	0.10
CRP (μg/ml)	1.4	1.39	1.45	1.35	0.35	0.01

NPY (ng/ml)	21.58	8.05	19.39	9.33	0.21	-0.42
anandamide (ng/ml)	4.79	4.04	4.56	3.53	0.20	-0.06
beta-endorphin (pg/ml)	3530.6	1198.07	4105.5	1508.76	0.0002	0.42
dynorphin (ng/ml)	136.52	47.56	150.75	56.18	0.009	0.27
leptin (pg/ml)	1794.87	1759.65	1619.6	1760.35	0.097	-0.10
oxytocin (pg/ml)	1038.87	1025.59	1102.5	1306.43	0.97	0.00

Supplementary Table 12: Commercial ELISA Kits

Target	Kit Model	Manufacturer
Oxytocin	ADI-900-153A	ENZO Life Sciences, Farmingdale NY, USA
Beta-endorphin	NBP2-78774	Novus Biologicals, Centennial CO, USA
Dynorphin	EIA-DYN	RayBiotech, Norcross GA, USA
Anandamide	MBS167217	MyBioSource, San Diego CA, USA
Cocaine and amphetamine regulated transcript (CART)	IHUCARTPTKT	Innovative Research, Novi MI, USA
C-reactive protein (CRP)	DCRP00B	USA R&D Systems, Minneapolis MN, USA
Neuropeptide Y (NPY)	EIA-NPY	RayBiotech, Norcross GA, USA

Supplementary Table 13: UPLC Metabolomic Detection Conditions

Component	Condition
Column	Acquity HSS T3 (2.1 mm x 100 mm, 1.8 μm)
Solvent A	Ultrapure water with 0.1% formic acid
Solvent B	Acetonitrile with 0.1% formic acid
Linear gradient elution	5% B to 90% B over 11 minutes
Flow rate	0.4 mL/min
Injection volume	2 μL

Supplementary Table 14: Metabolomic Detection Mass Spectrum Conditions

Parameter	ESI+	ESI-
Curtain Gas (psi)	25	25
IonSpray Voltage (V)	5500	4500
Temperature (°C)	500	500
Ion Source Gas 1 (psi)	50	50
Ion Source Gas 2 (psi)	50	50
Declustering Potential	80	-80
Collision Energy	30	-30
Collision Energy Spread	15	15
Collision Gas (CAD) (widely targeted phase)	High	High

Instrument tuning and mass calibration were performed with 10 and 100 μmol/L polypropylene glycol solutions in QQQ and LIT modes, respectively. A specific set of MRM transitions were monitored for each period according to the metabolites eluted within that period.

Supplementary Tables 15 A-G: A Priori Protein Index Components

15A. Brain-Derived Neurotrophic Factor (BDNF) Pathway Index

Entrez Gene Symbol	Target Name	UniProt	Entrez Gene ID	Dilution
RAF1	RAF proto-oncogene serine/threonine-protein kinase	P04049	5894	20
JUN	Transcription factor AP-1	P05412	3725	20
NRAS	GTPase NRas	P01111	4893	20
SLITRK1	SLIT and NTRK-like protein 1	Q96PX8	114798	0.5
PTPN11	Tyrosine-protein phosphatase non-receptor type 11	Q06124	5781	20
BDNF	Brain-derived neurotrophic factor	P23560	627	0.5
SLITRK1	SLIT and NTRK-like protein 1	Q96PX8	114798	20
AKT1	RAC-alpha serine/threonine-protein kinase	P31749	207	20
BRAF	Serine/threonine-protein kinase B-raf	P15056	673	20
SHC1	SHC-transforming protein 1:Phosphotyrosine Interaction Domain	P29353	6464	20
HRAS	GTPase HRas	P01112	3265	0.5
MECP2	Methyl-CpG-binding protein 2	P51608	4204	20
KRAS	GTPase KRas	P01116	3845	20
CREB1	cAMP response element-binding protein	P16220	1385	20

GSK3B	Glycogen synthase kinase-3 beta	P49841	2932	20
BDNF	Brain-derived neurotrophic factor	P23560	627	20
MAPK3	Mitogen-activated protein kinase 3	P27361	5595	20
AKT1	RAC-alpha serine/threonine-protein kinase	P31749	207	20
RAC1	Ras-related C3 botulinum toxin substrate 1	P63000	5879	0.5
MAPK1	Mitogen-activated protein kinase 1	P28482	5594	20
GSK3B	Glycogen synthase kinase-3 beta	P49841	2932	20
CAMK2A	Calcium/calmodulin-dependent protein kinase type II subunit alpha	Q9UQM7	815	20
CAMK2B	Calcium/calmodulin-dependent protein kinase type II subunit beta	Q13554	816	20
PTPN11	Tyrosine-protein phosphatase non-receptor type 11	Q06124	5781	20
NTRK1	High affinity nerve growth factor receptor	P04629	4914	20
NTF4	Neurotrophin-4	P34130	4909	20
NTRK2	BDNF/NT-3 growth factors receptor	Q16620	4915	20
KRAS	GTPase KRas	P01116	3845	20
SHC1	SHC-transforming protein 1:Src Homology domain	P29353	6464	20
GRB2	Growth factor receptor-bound protein 2	P62993	2885	20
DLG4	Disks large homolog 4	P78352	1742	20
NGFR	Tumor necrosis factor receptor superfamily member 16	P08138	4804	20
PIK3R1	Phosphatidylinositol 3-kinase regulatory subunit alpha	P27986	5295	20
SOS1	Son of sevenless homolog 1	Q07889	6654	20

15B. SCFA Index

**Entrez Gene Symbol**	**Target Full Name**	**UniProt**	**Entrez Gene ID**	**Dilution**
SLC5A8	Sodium-coupled monocarboxylate transporter 1	Q8N695	160728	0.5
LDHA	L-lactate dehydrogenase A chain	P00338	3939	0.5
ACADS	Short-chain specific acyl-CoA dehydrogenase; mitochondrial	P16219	35	20
ACOX1	Peroxisomal acyl-coenzyme A oxidase 1	Q15067	51	20
ACSS2	Acetyl-coenzyme A synthetase; cytoplasmic	Q9NR19	55902	20
LDHB	L-lactate dehydrogenase B chain	P07195	3945	0.005
FASN	Fatty acid synthase	P49327	2194	20

LDHA	L-lactate dehydrogenase A chain	P00338	3939	0.5

15C. Neuronal Index

Entrez Gene Symbol	Target Name	UniProt	Entrez Gene ID	Dilution
SIRT5	NAD-dependent protein deacylase sirtuin-5; mitochondrial	Q9NXA8	23408	20
CREBBP	CREB-binding protein	Q92793	1387	0.5
RBBP4	Histone-binding protein RBBP4	Q09028	5928	20
SIRT3	NAD-dependent protein deacetylase sirtuin-3; mitochondrial	Q9NTG7	23410	20
HDAC2	Histone deacetylase 2	Q92769	3066	20
SIRT1	NAD-dependent protein deacetylase sirtuin-1	Q96EB6	23411	20
SIRT6	silent mating type information regulation 2 homolog	Q8N6T7	51548	20
SIRT2	NAD-dependent protein deacetylase sirtuin-2	Q8IXJ6	22933	20
MEF2D	Myocyte-specific enhancer factor 2D	Q14814	4209	20
HDAC6	Histone deacetylase 6	Q9UBN7	10013	20
HDAC4	Histone deacetylase 4	P56524	9759	20
HDAC8	Histone deacetylase 8	Q9BY41	55869	20
SIRT2	NAD-dependent protein deacetylase sirtuin-2	Q8IXJ6	22933	0.5
MEF2C	Myocyte-specific enhancer factor 2C	Q06413	4208	20

15D. Glycolysis Pathway Index

Entrez Gene Symbol	Target Name	UniProt	Entrez Gene ID	Dilution
ENO2	Gamma-enolase	P09104	2026	20
ENO2	Gamma-enolase	P09104	2026	20
ENO1	Alpha-enolase	P06733	2023	0.5
HK2	Hexokinase-2	P52789	3099	20
HK1	Hexokinase-1	P19367	3098	20
LDHA	L-lactate dehydrogenase A chain	P00338	3939	0.5

ENO3	Beta-enolase	P13929	2027	20
PFKM	ATP-dependent 6-phosphofructokinase; muscle type	P08237	5213	0.5
ALDOB	Fructose-bisphosphate aldolase B	P05062	229	0.5
HK3	Hexokinase-3	P52790	3101	20
GAPDH	Glyceraldehyde-3-phosphate dehydrogenase	P04406	2597	0.005
LDHB	L-lactate dehydrogenase B chain	P07195	3945	0.005
PGAM1	Phosphoglycerate mutase 1	P18669	5223	20
PKM	Pyruvate kinase PKM	P14618	5315	0.5
GPI	Glucose-6-phosphate isomerase	P06744	2821	20
TPI1	Triosephosphate isomerase	P60174	7167	0.5
PGK1	Phosphoglycerate kinase 1	P00558	5230	20
ALDOA	Fructose-bisphosphate aldolase A	P04075	226	0.005
LDHA	L-lactate dehydrogenase A chain	P00338	3939	0.5
LDHC	L-lactate dehydrogenase C chain	P07864	3948	20
ALDOC	Fructose-bisphosphate aldolase C	P09972	230	0.5

15E. Endogenous Opioid Pathway Index

**Entrez Gene Symbol**	**Target Name**	**UniProt**	**Entrez Gene ID**	**Dilution**
GABBR1	Gamma-aminobutyric acid type B receptor subunit 1	Q9UBS5	2550	20
GNAI3	Guanine nucleotide-binding protein G(k) subunit alpha	P08754	2773	20
GABBR2	Gamma-aminobutyric acid type B receptor subunit 2:Extracellular domain	O75899	9568	20
PNOC	Prepronociceptin	Q13519	5368	20
GNAI1	Guanine nucleotide-binding protein G(i) subunit alpha-1	P63096	2770	20
PDYN	Dynorphin A (1-17)	P01213	5173	20
CREB1	cAMP response element-binding protein	P16220	1385	20
POMC	Beta-endorphin	P01189	5443	20
CAMK2A	Calcium/calmodulin-dependent protein kinase type II subunit alpha	Q9UQM7	815	20
CAMK2B	Calcium/calmodulin-dependent protein kinase type II subunit beta	Q13554	816	20
PRKACA	cAMP-dependent protein kinase catalytic subunit alpha	P17612	5566	20

POMC	Corticotropin	P01189	5443	20
PENK	Proenkephalin-A	P01210	5179	20
POMC	Pro-opiomelanocortin	P01189	5443	20
GABBR2	Gamma-aminobutyric acid type B receptor subunit 2:Cytoplasmic domain	O75899	9568	20

15F. Pro-Inflammatory Pathways Index

Entrez Gene Symbol	Target Name	UniProt	Entrez Gene ID	Dilution
STAT3	Signal transducer and activator of transcription 3	P40763	6774	20
STAT3	Signal transducer and activator of transcription 3	P40763	6774	20
STAT1	Signal transducer and activator of transcription 1-alpha/beta	P42224	6772	20
STAT6	Signal transducer and activator of transcription 6	P42226	6778	20
TLR4	Toll-like receptor 4	O00206	7099	20
STAT1	Signal transducer and activator of transcription 1-alpha/beta	P42224	6772	20
IL10	Interleukin-10	P22301	3586	20
IL12B	Interleukin-12 subunit beta	P29460	3593	20
IFNG	Interferon gamma	P01579	3458	20
IRAK4	Interleukin-1 receptor-associated kinase 4	Q9NWZ3	51135	20
S100A8	Calgranulin A	P05109	6279	20
S100A8	Calgranulin A	P05109	6279	20
CCL5	C-C motif chemokine 5	P13501	6352	20
IL6	Interleukin-6	P05231	3569	20
CCL2	C-C motif chemokine 2	P13500	6347	20
IL10	Interleukin-10	P22301	3586	20
MAPK3	Mitogen-activated protein kinase 3	P27361	5595	20
IFNG	Interferon gamma	P01579	3458	20
IL1B	Interleukin-1 beta	P01584	3553	20
MAPK1	Mitogen-activated protein kinase 1	P28482	5594	20
CXCL8	Interleukin-8	P10145	3576	20
IL17A	Interleukin-17A	Q16552	3605	20
PTGS2	Prostaglandin G/H synthase 2	P35354	5743	20
TLR2	Toll-like receptor 2	O60603	7097	20

IL6	Interleukin-6	P05231	3569	20
S100A9	Protein S100-A9	P06702	6280	20
CCL5	C-C motif chemokine 5	P13501	6352	0.5
IL18	Interleukin-18	Q14116	3606	20
TNF	Tumor necrosis factor	P01375	7124	20
TNF	Tumor necrosis factor	P01375	7124	20
S100A9	Protein S100-A9	P06702	6280	20
IL17A	Interleukin-17A	Q16552	3605	20
NFKB1	Nuclear factor NF-kappa-B p105 subunit	P19838	4790	20

15G. Anti-Inflammatory Pathways Index

Entrez Gene Symbol	Target Name	UniProt	Entrez Gene ID	Dilution
STAT6	Signal transducer and activator of transcription 6	P42226	6778	20
MSR1	Macrophage scavenger receptor types I and II:Cytoplasmic domain	P21757	4481	20
MSR1	Macrophage scavenger receptor types I and II:Extracellular domain	P21757	4481	20
SOCS3	Suppressor of cytokine signaling 3	O14543	9021	0.5
IL4	Interleukin-4	P05112	3565	20
IL10	Interleukin-10	P22301	3586	20
TNFAIP3	Tumor necrosis factor alpha-induced protein 3	P21580	7128	20
IL13	Interleukin-13	P35225	3596	20
TGFB1	Transforming growth factor beta-1	P01137	7040	20
MSR1	Macrophage scavenger receptor types I and II:Extracellular domain	P21757	4481	20
CEBPB	CCAAT/enhancer-binding protein beta	P17676	1051	20
STAT5B	Signal transducer and activator of transcription 5B	P51692	6777	20
HMOX1	Heme oxygenase 1	P09601	3162	0.5
KLF4	Krueppel-like factor 4	O43474	9314	20
NFKBIA	NF-kappa-B inhibitor alpha	P25963	4792	20
STAT5A	Signal transducer and activator of transcription 5A	P42229	6776	20
TGFB1	Transforming growth factor beta-1	P01137	7040	20
FOXP3	Forkhead box protein P3	Q9BZS1	50943	20

CEBPB	CCAAT/enhancer-binding protein beta	P17676	1051	20
TGFB1	Transforming growth factor beta-1	P01137	7040	20
PPARG	Peroxisome proliferator activated receptor gamma	P37231	5468	20
MRC1	Macrophage mannose receptor 1	P22897	4360	0.5
IL10	Interleukin-10	P22301	3586	20
IL4	Interleukin-4	P05112	3565	20
IL13	Interleukin-13	P35225	3596	20
MSR1	Macrophage scavenger receptor types I and II:Extracellular domain	P21757	4481	20
CD274	Programmed cell death 1 ligand 1	Q9NZQ7	29126	20
IL1RN	Interleukin-1 receptor antagonist protein	P18510	3557	20
ARG1	Arginase-1	P05089	383	20

15H. Oxidative Phorphorylation Index

Entrez Gene Symbol	Target Name	UniProt	Entrez Gene ID	Dilution
COX5A	Cytochrome c oxidase subunit 5A (mitochondrial)	P20674	9377	20
NDUFA2	NADH dehydrogenase 1 alpha subcomplex subunit 2	O43678	4695	20
ATP5F1B	ATP synthase subunit beta (mitochondrial)	P06576	506	20
ATP5PF	ATP synthase-coupling factor 6 (mitochondrial)	P18859	522	20
ATP5PO	ATP synthase subunit O (mitochondrial)	P48047	539	20
COX7A2L	Cytochrome c oxidase subunit 7A-related protein	O14548	9167	20

Supplementary Table 16. Metabolomic Pathway Analysis

	Total cmpds	Hits	Raw *p*	-log10(*p*)	Holm adjust	FDR	Impact
Tryptophan metabolism	41	6	0.00	3.20	0.03	0.03	0.28
Retinol metabolism	17	1	0.01	1.98	0.54	0.25	0.12
Sphingolipid metabolism	32	3	0.01	1.84	0.74	0.25	0.02
Biosynthesis of unsaturated fatty acids	36	1	0.04	1.44	1	0.35	0.00
Starch and sucrose metabolism	18	1	0.04	1.40	1	0.35	0.42

Neomycin, kanamycin and gentamicin biosynthesis	2	1	0.04	1.40	1	0.35	0.00
Glycerophospholipid metabolism	36	2	0.06	1.20	1	0.48	0.04
Arginine and proline metabolism	36	6	0.08	1.12	1	0.51	0.31
Steroid hormone biosynthesis	87	8	0.10	1.02	1	0.52	0.17
Nicotinate and nicotinamide metabolism	15	4	0.10	1.01	1	0.52	0.19
Drug metabolism - other enzymes	39	1	0.14	0.85	1	0.59	0.00
One carbon pool by folate	9	2	0.14	0.84	1	0.59	0.00
Folate biosynthesis	27	1	0.14	0.84	1	0.59	0.00
Amino sugar and nucleotide sugar metabolism	42	3	0.17	0.77	1	0.65	0.01
Porphyrin metabolism	31	2	0.21	0.69	1	0.69	0.07
Glutathione metabolism	28	4	0.22	0.66	1	0.69	0.05
Inositol phosphate metabolism	30	1	0.22	0.65	1	0.69	0.13
Purine metabolism	70	4	0.28	0.56	1	0.69	0.07
Ether lipid metabolism	20	1	0.28	0.55	1	0.69	0.04
Cysteine and methionine metabolism	33	7	0.29	0.54	1	0.69	0.31
Glycine, serine and threonine metabolism	33	5	0.30	0.53	1	0.69	0.34
Lipoic acid metabolism	28	1	0.33	0.48	1	0.69	0.00
Histidine metabolism	16	5	0.33	0.48	1	0.69	0.22
Butanoate metabolism	15	4	0.34	0.47	1	0.69	0.00
Fatty acid biosynthesis	47	1	0.34	0.46	1	0.69	0.00
Lysine degradation	30	1	0.36	0.45	1	0.69	0.11
Propanoate metabolism	22	3	0.37	0.43	1	0.69	0.00
Tyrosine metabolism	42	4	0.38	0.42	1	0.69	0.25
Phenylalanine metabolism	8	4	0.40	0.40	1	0.69	0.60
Nitrogen metabolism	6	2	0.41	0.39	1	0.69	0.00
Ascorbate and aldarate metabolism	9	2	0.41	0.39	1	0.69	0.00
Pentose phosphate pathway	23	4	0.42	0.38	1	0.69	0.18
Fructose and mannose metabolism	20	3	0.46	0.34	1	0.73	0.03
Citrate cycle (TCA cycle)	20	5	0.47	0.33	1	0.73	0.28
Glycerolipid metabolism	16	1	0.53	0.28	1	0.79	0.09
Taurine and hypotaurine metabolism	8	2	0.54	0.27	1	0.79	0.60
Glyoxylate and dicarboxylate metabolism	32	6	0.58	0.24	1	0.81	0.18
Galactose metabolism	27	5	0.59	0.23	1	0.81	0.03
Phenylalanine, tyrosine and tryptophan biosynthesis	4	2	0.60	0.22	1	0.81	1.00

Ubiquinone and other terpenoid-quinone biosynthesis	18	1	0.66	0.18	1	0.86	0.00
Vitamin B6 metabolism	9	1	0.67	0.17	1	0.86	0.00
Arginine biosynthesis	14	8	0.71	0.15	1	0.86	0.57
Linoleic acid metabolism	5	1	0.71	0.15	1	0.86	0.00
beta-Alanine metabolism	21	4	0.71	0.15	1	0.86	0.00
Valine, leucine and isoleucine biosynthesis	8	2	0.74	0.13	1	0.87	0.00
Primary bile acid biosynthesis	46	5	0.82	0.09	1	0.90	0.03
D-Amino acid metabolism	15	1	0.82	0.08	1	0.90	0.00
Valine, leucine and isoleucine degradation	40	3	0.84	0.08	1	0.90	0.03
Alanine, aspartate and glutamate metabolism	28	9	0.84	0.08	1	0.90	0.58
Pentose and glucuronate interconversions	19	1	0.85	0.07	1	0.90	0.07
Pyrimidine metabolism	39	7	0.88	0.06	1	0.91	0.22
Pantothenate and CoA biosynthesis	20	4	0.96	0.02	1	0.97	0.01
Caffeine metabolism	10	9	0.97	0.01	1	0.97	1.00

Supplementary Table 17. exRNAs

(RNAseq Excel file)

Supplementary Figures

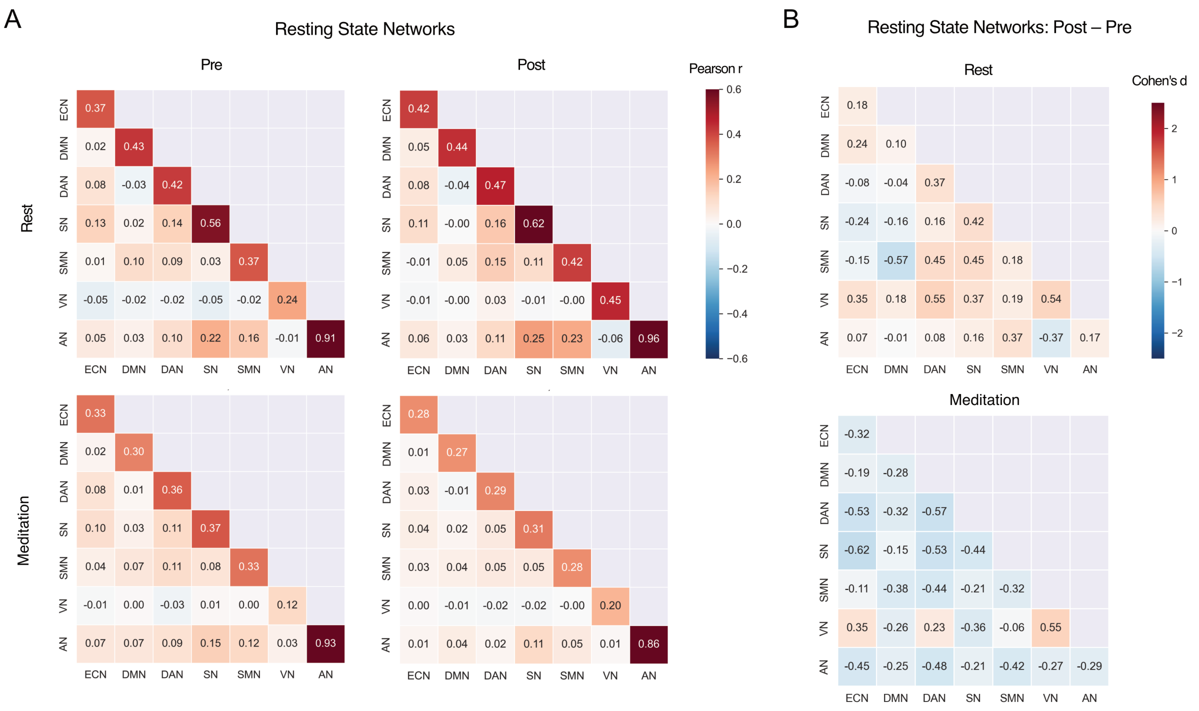

Supplementary Figure 1

A. Resting State Functional Connectivity Networks. Within- and between-network resting-state connectivity during rest and meditation, pre- and post-intervention. Cell values and color scale represent mean Pearson correlation values across participants. As expected, intra-network connectivity is stronger than inter-network connectivity, validating data quality.

B. Pre Versus Post-Intervention Changes in Functional Connectivity. Pre- versus post-retreat resting state network connectivity during (Left) rest and (Right) meditation. Cell values and color scale represent Cohen's d effect size values from paired samples t-tests. No statistically significant results adjusted for 28 multiple comparisons, $p_{FWE} < 0.05$; however, pre-to-post intervention there is generally weaker intra and inter-network connectivity during meditation (with exceptions clustered around the visual network), and generally stronger connectivity during rest, except for SMN-DMN, SMN-ECN, SN-ECN, SN-DMN, and AN-VN.

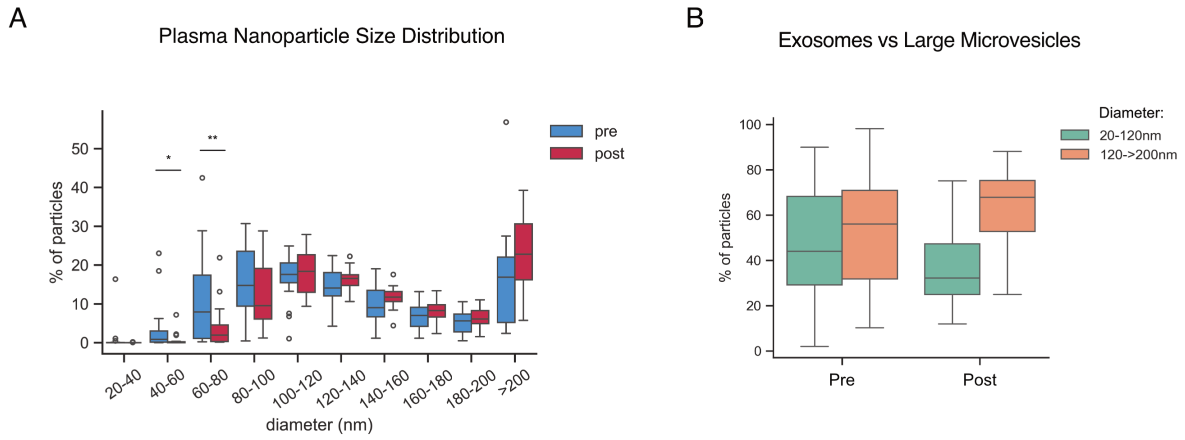

Supplementary Figure 2

A. Plasma Nanoparticle Size Distributions. Asterisks denote statistical significance for pre vs post two-tailed paired t-tests: * ($p < 0.05$), ** ($p < 0.01$).

B. Exosomes Versus Larger Microvesicles. Percentage of plasma exosomes (20-120nm) versus larger microvesicles (120-200nm) pre- and post-intervention. Asterisks denote statistical significance on two-tailed t-tests, * ($p < 0.05$), ** ($p < 0.01$), *** ($p < 0.001$).

g:Profiler Proteomic Enrichment Analysis

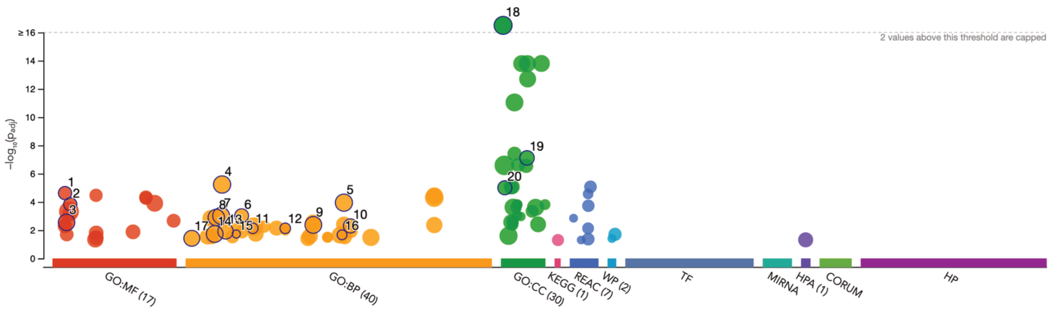

ID	Source	Term ID	Term Name	p_{adj} (query_1)
1	GO:MF	GO:0004866	endopeptidase inhibitor activity	2.365×10^{-5}
2	GO:MF	GO:0008201	heparin binding	1.458×10^{-4}
3	GO:MF	GO:0005102	signaling receptor binding	2.788×10^{-3}
4	GO:BP	GO:0009056	catabolic process	5.828×10^{-6}
5	GO:BP	GO:0051246	regulation of protein metabolic process	1.126×10^{-4}
6	GO:BP	GO:0015850	organic hydroxy compound transport	9.465×10^{-4}
7	GO:BP	GO:0008283	cell population proliferation	9.552×10^{-4}
8	GO:BP	GO:0007155	cell adhesion	1.252×10^{-3}
9	GO:BP	GO:0044281	small molecule metabolic process	4.223×10^{-3}
10	GO:BP	GO:0052548	regulation of endopeptidase activity	4.982×10^{-3}
11	GO:BP	GO:0019433	triglyceride catabolic process	7.309×10^{-3}
12	GO:BP	GO:0034368	protein-lipid complex remodeling	7.309×10^{-3}
13	GO:BP	GO:0009617	response to bacterium	1.200×10^{-2}
14	GO:BP	GO:0006952	defense response	1.859×10^{-2}
15	GO:BP	GO:0010901	regulation of very-low-density lipoprotein parti...	1.865×10^{-2}
16	GO:BP	GO:0051004	regulation of lipoprotein lipase activity	2.127×10^{-2}
17	GO:BP	GO:0001932	regulation of protein phosphorylation	3.759×10^{-2}
18	GO:CC	GO:0005576	extracellular region	9.894×10^{-28}
19	GO:CC	GO:0062023	collagen-containing extracellular matrix	7.626×10^{-8}
20	GO:CC	GO:0005788	endoplasmic reticulum lumen	9.936×10^{-6}

**version**	e109_eg56_p17_1d3191d
**date**	8/31/2023, 1:26:36 PM
**organism**	hsapiens

g:Profiler

Supplementary Figure 3. Results of a proteomic enrichment analysis using g:Profiler. The top panel shows a multi-category Manhattan plot where significant gene ontology (GO) terms, pathways, and protein complexes are visualized. Each dot represents an enriched term, with the x-axis indicating the category (e.g., GO: Molecular Function [MF], GO: Biological Process [BP], KEGG pathways, Reactome, CORUM) and the y-axis showing the -log10 adjusted p-value (p_{adj}) of enrichment. Terms with $p_{adj} < 0.05$ are considered significant. Two values exceed the threshold and are capped at the top of the plot. The bottom table lists the top 20 enriched terms, including their source, term ID, term name, and adjusted p-value. The most enriched terms include "extracellular region" ($p_{adj} = 9.894 \times 10^{-28}$), "collagen-containing extracellular matrix" ($p_{adj} = 7.626 \times 10^{-8}$), and "endoplasmic reticulum lumen" ($p_{adj} = 9.936 \times 10^{-6}$). The analysis highlights key molecular functions, biological processes, and cellular components relevant to the proteomic dataset under investigation.

Heat Map Representation of Enriched Biological Pathways

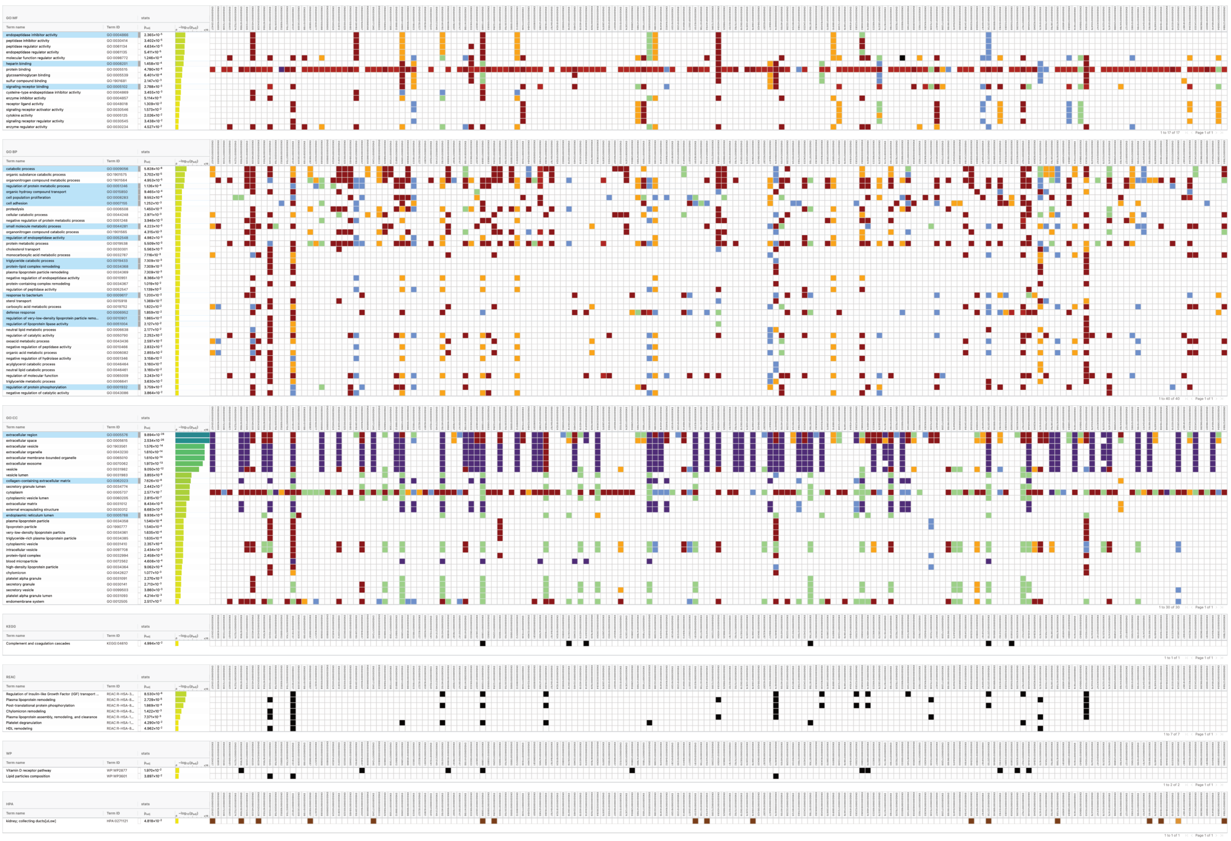

Supplementary Figure 4. Heatmap Representation of Enriched Biological Pathways
The heatmap depicts the enrichment of various biological pathways and functional terms across multiple samples using g:Profiler analysis. The y-axis lists the enriched terms grouped by categories, including Gene Ontology: Molecular Function (GO:MF), Biological Process (GO:BP), Cellular Component (GO:CC), KEGG pathways, Reactome pathways, CORUM complexes, and transcription factors (TF). The x-axis represents individual samples. Each colored cell indicates the presence and significance of a specific term in a corresponding sample, with color intensity reflecting the level of enrichment (adjusted p-value). Darker colors indicate stronger enrichment (lower p-values). Terms that are significantly enriched (p_{adj} < 0.05) are highlighted, emphasizing distinct biological processes and cellular functions enriched in subsets of the dataset. The top-ranked terms in each category, such as "endocytic vesicle membrane," "lipid catabolic process," and "extracellular matrix," are shown alongside a bar plot indicating their relative significance across the dataset. This visualization highlights key differences in functional enrichment across samples, providing insights into the underlying biological variations.

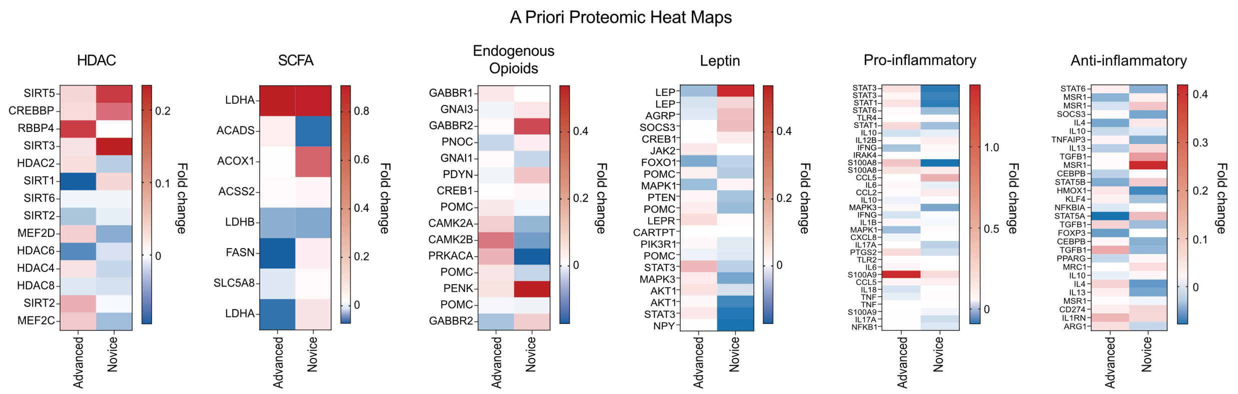

Supplementary Figure 5. Proteomic *A Priori* Protein Heat Maps. Heat maps for a priori protein indices showing fold change levels per protein for advanced and novice participants.

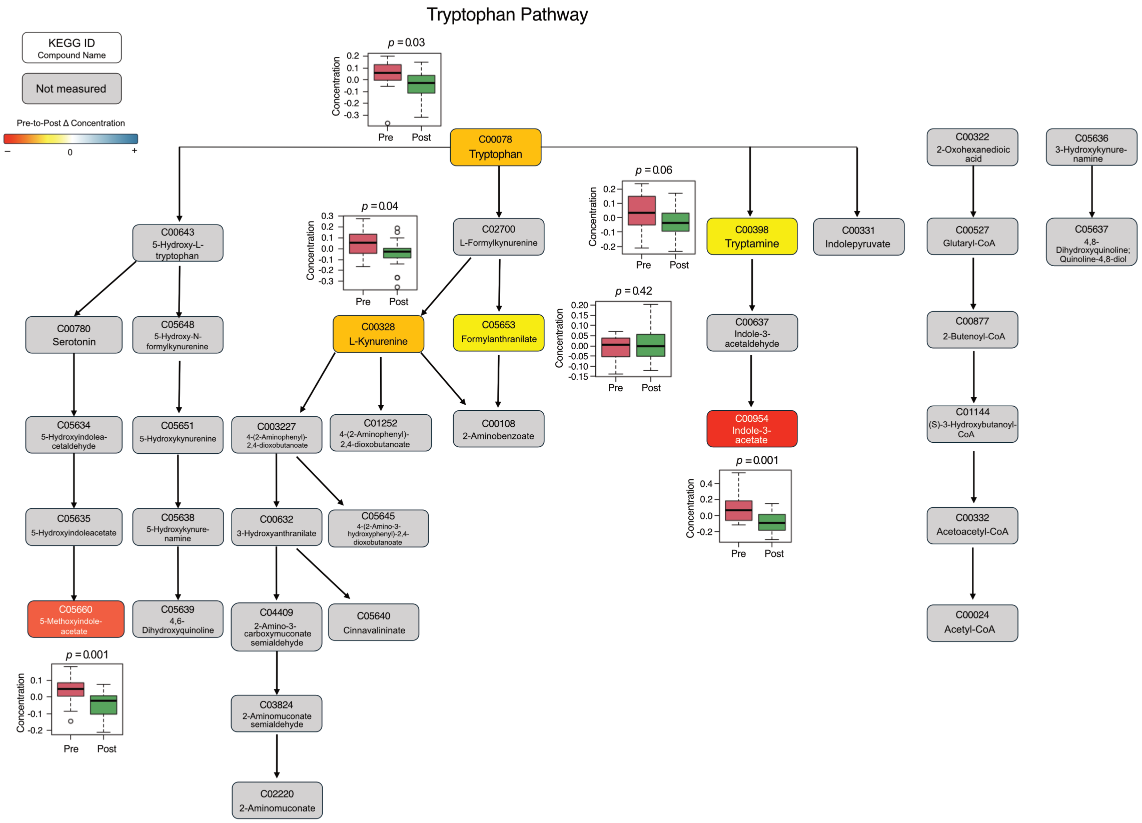

Supplementary Figure 6. Full Tryptophan Pathway. Colored boxes represent detected metabolites. Color scale represents the magnitude of pre-to-post change in concentration. Box plots show pre and post concentration distributions and two-tailed paired t-test p-values. The pathway is significantly downregulated.
