# Supplementary material for: Neural and molecular changes during a mind-body reconceptualization, meditation, and open label placebo healing retreat: an observational study
Source: Commun Biol. 2025 Nov 6;8:1525. doi: 10.1038/s42003-025-09088-3 (PMC12592435; doi:10.1038/s42003-025-09088-3)
Supplement: Supplementary file 3 — Description of Additional Supplementary Files [file 42003_2025_9088_MOESM3_ESM.pdf]

Description of Additional Supplementary files

File name: Supplementary Data 1

Description: Values for all data points in plots and reported mean value.
